# High Airway‐To‐Vessel Volume Ratio and Visual Bronchiectasis Are Associated With Exacerbations in COPD

**DOI:** 10.1111/resp.70114

**Published:** 2025-08-31

**Authors:** Nobuyasu Wakazono, Kaoruko Shimizu, Naoya Tanabe, Akira Oguma, Hironi Makita, Kazufumi Okada, Miho Wakazono, Hiroki Nishimura, Yuichi Kojima, Michiko Takimoto‐Sato, Munehiro Matsumoto, Yuki Abe, Ayako Igarashi‐Sugimoto, Nozomu Takei, Hirokazu Kimura, Houman Goudarzi, Takeshi Hattori, Ichizo Tsujino, Susumu Sato, Shigeo Muro, Masaharu Nishimura, Toyohiro Hirai, Satoshi Konno

**Affiliations:** ^1^ Department of Respiratory Medicine, Faculty of Medicine Hokkaido University Sapporo Japan; ^2^ Department of Respiratory Medicine, Graduate School of Medicine Kyoto University Kyoto Japan; ^3^ Hokkaido Medical Research Institute of Respiratory Diseases Sapporo Japan; ^4^ Data Science Center, Promotion Unit, Institute of Health Science Innovation for Medical Care Hokkaido University Hospital Sapporo Japan; ^5^ Division of Pulmonary, Critical Care and Sleep Medicine, Department of Medicine Icahn School of Medicine at Mount Sinai New York New York USA; ^6^ Department of Respiratory Medicine National Hospital Organization Hokkaido Medical Center Sapporo Japan; ^7^ Department of Respiratory Medicine Nara Medical University Kashihara Japan

**Keywords:** bronchiectasis, chronic obstructive pulmonary disease, computed tomography, exacerbation, pulmonary blood vessel

## Abstract

**Background and Objective:**

The effects of the volume mismatch between the airway and lung vasculature on exacerbation in chronic obstructive pulmonary disease (COPD) is uncertain. We aimed to examine the association between an increased volume ratio of the airway to lung blood vessels (AVR) and exacerbations, regardless of visually assessed bronchiectasis (modified Reiff [mReiff] score) and extrapulmonary vasculature on computed tomography (CT), in patients with COPD during a 5‐year follow‐up period.

**Methods:**

Participants were recruited from the Hokkaido COPD Cohort Study (original, *N* = 96) and Kyoto University cohort (validation, *N* = 130). CT‐derived indices of the airway and vasculature, mReiff scores, and ratio of pulmonary artery diameter to aorta diameter (PA/Ao) were evaluated. The Kaplan–Meier method with log‐rank tests was used to compare the high (highest quartile) and low (other quartiles) groups, while multivariable Cox proportional hazards models explored the factors associated with the time to first exacerbation.

**Results:**

The high AVR group showed a shorter time to first exacerbation than the low AVR group in analyses of both all patients and those without visual bronchiectasis. High AVR was significantly associated with exacerbations [Hazard ratio [95% confidence interval]: original, 3.85 [1.17, 12.6]; validation, 2.01 [1.15, 3.52]), irrespective of mReiff scores and PA/Ao in all patients. The lung‐volume‐corrected airway or blood vessel volumes did not correlate with the time to first exacerbation.

**Conclusion:**

High AVR was associated with a shorter time to first exacerbation, complementary to mReiff score and PA/Ao, suggesting that AVR is a novel CT‐derived predictor of exacerbation in COPD.

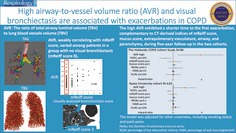

## Graphical abstract

This study demonstrated the association between an unbalanced volume ratio of airway to pulmonary blood vessels and shorter time to the first exacerbation in patients with chronic obstructive pulmonary disease, complementary to the presence of visually diagnosed bronchiectasis.

**See related** editorial

## 1 Introduction

Chronic obstructive pulmonary disease (COPD) is a socioeconomic burden owing to its high prevalence, progressive nature, and various comorbidities; additionally, it is the third leading cause of death worldwide [1]. Specifically, exacerbation requires hospitalisation and emergency visits and is possibly followed by a deterioration in activities of daily living, reduced lung function, and physical impairment [2, 3]. Thus, identification of the aetiology of exacerbations and of subgroups with a high risk of frequent exacerbations is needed for better management of patients with COPD.

Studies have shown that air pollution [4], infections [5], high blood eosinophil counts [6], and a history of exacerbation [7] are associated with COPD exacerbation. Furthermore, radiological features, such as emphysema [8, 9] and bronchiectasis [10, 11, 12], on computed tomography (CT), play a role in subtyping for identifying a high risk of exacerbation. Notably, extrapulmonary vascular morphological measures, such as an enlargement of the pulmonary artery diameter, have been reported as predictors of future exacerbations and mortality [13].

Bronchiectasis is defined as permanent and progressive dilation of the airways via inflammation, infection, and repair of the bronchial mucosa [14], which is related to exacerbation in patients with COPD [10, 15] and is possibly reflective of the triad of greater bronchial inflammation, severe exacerbations, and more frequent colonisation of the bronchial mucosa by potentially pathogenic microorganisms [10, 16].

Naidich et al. defined CT‐based visual bronchiectasis as (I) the lack of tapering of the bronchi, (II) dilation of the bronchi when the internal diameter is larger than that of the adjacent pulmonary artery, or (III) visualisation of the peripheral bronchi within 1 cm of the costal pleural surface or adjacent mediastinal pleural surface [17]. Bhalla and Reiff proposed scoring systems considering the severity and degree of bronchiectasis progression [18, 19]. Visual assessments have focused on regional bronchiectasis, which does not always result in functional impairment. However, a mismatch between airway and pulmonary blood vessels volume may contribute to poor clinical outcomes, such as exacerbation, through VQ mismatch. We, therefore, hypothesised that this volume mismatch would be associated with exacerbations, complementary to visually assessed bronchiectasis in patients with COPD.

This study aimed to examine whether the volume mismatch between the airway and lung vessel volume, expressed as the volume ratio of the airway to lung blood vessels (AVR), assessed as the ratio of total airway luminal volume (TAV) to total blood vessel volume (TBV) on CT, was associated with a shorter time to the first exacerbation during a 5‐year follow‐up period, regardless of visually assessed bronchiectasis and the ratio of the pulmonary artery diameter to the aorta diameter (PA/Ao), in two independent prospective Japanese cohorts.

## 2 Methods

Details of the two cohorts are presented as Supporting Information.

### 2.1 Participants

Participants were recruited from the Hokkaido COPD Cohort Study (original cohort) [20, 21] and Kyoto University cohort (validation cohort) [22]. Both studies were performed according to the revised Declaration of Helsinki. The Institutional Review Board (IRB) of Hokkaido University Hospital approved this study (reference number: 018‐0394). All participants provided written informed consent according to the Hokkaido COPD Cohort Protocol. Additionally, we informed patients about the study protocol via the Internet and provided an opt‐out option, in compliance with the IRB of Hokkaido University Hospital. Details of the two cohorts are presented as Supporting Information.

### 2.2 Clinical Indices and Outcomes

Exacerbation was defined using the medical definition: meeting the symptomatic criteria plus requiring treatment with antibiotics or systemic steroids or hospital admission [20, 23, 24]. Exacerbation incidence was tracked using information collected via postcards (response rate, > 99%). Data were collected every month for 5 years from baseline. Detailed information is presented as Supporting Information.

### 2.3 Quantitative Chest CT

In the original cohort, CT data were analysed at full inspiration using a 1.25‐mm slice thickness with a Somatom plus Volume Zoom scanner (Siemens AG, Berlin, Germany) at 140 kVp and 150 mA, and images were reconstructed using a soft algorithm (standard kernel, B30f; sharp kernel, B60f) [9]. In the validation cohort, CT data were analysed at full inspiration using a 0.5‐mm slice thickness with an Aquilion 64 scanner (Toshiba, Tokyo, Japan) at 120 kVp with auto‐exposure control, and images were reconstructed using a sharp algorithm (FC56) (Table S1). Detailed information is presented as Supporting Information.

### 2.4 CT Imaging Assessment

#### 2.4.1 Intrapulmonary Indices

We performed CT analysis using AVIEW (Coreline Soft Inc., Seoul, South Korea). The airway tree was constructed by extending the original images of airways extracted using a threshold of −950 Hounsfield unit (HU) through an artificial‐intelligence‐based methodology [25]. TAV was defined as the total volume of the intrapulmonary airway in the lungs. We used the ratio of low attenuation volume below −950 HU to the lung volume (LV) on CT (%LAV) as an index for emphysema. The percentage of wall area (%WA), defined as wall area (WA) divided by the sum of WA and airway luminal area (LA), was averaged between the right apical (RB1) and lateral basal (RB8) segmental airways (3rd generation). LA and WA were automatically measured in the central third of each branch. The Beyond Frangi algorithm was applied to enhance blood vessel extraction [26, 27]. CT data were binarized using a −750 HU threshold. Vessel volume was quantified in AVIEW by multiplying the number of vessel‐classified voxels by voxel size, using a three‐dimensional binary mask. Vessels within the masked lungs were included in the TBV [26, 27, 28]. BV5 was defined as an aggregate blood vessel volume of < 5 mm^2^ in the lungs. The mucus score was obtained by visual inspection of all the airway segments, performed independently by two pulmonologists [29].

#### 2.4.2 Visual Bronchiectasis Assessment

The modified Reiff (mReiff) score was employed to assess bronchiectasis quantitatively. The mReiff score ranges from 0 to 1, based on the number of affected lobes (maximum: 6, including the lingula) and the severity of bronchial dilatation compared to the adjacent blood vessels (0 = no bronchiectasis, 1 = 1–2 times, 2 = 2–3 times, 3 = over 3 times) [30].

### 2.5 Pulmonary Function Tests

Detailed information is presented as Supporting Information.

### 2.6 Statistical Analysis

Given the hypothesis that higher AVR has a stronger effect on exacerbation, we explored different cut‐off values of AVR, resulting in significant differences between high and low AVR groups in the 25th, 20th, and 12.5th percentiles in the original cohort and the 25th and 20th percentiles in the validation cohort. Subsequently, we adopted the 25th percentiles for the AVR cut‐off in both cohorts. Moreover, the values of the third quartile of TAV corrected by lung volume (LV) (TAV/LV) and first quartile of TBV corrected by LV (TBV/LV) were utilised to divide participants in the original cohort. Relationships between mReiff scores and CT indices, including AVR, were analysed using Spearman's test. AVR for each Global Initiative for Chronic Obstructive Lung Disease (GOLD) classification was analysed using Steel's test. Exacerbation‐free survival was compared between participants with high and low AVR, TAV/LV, and TBV/LV using the Kaplan–Meier method and log‐rank test. Multivariate Cox proportional hazards models were employed to assess the impact of covariates, including AVR, on the time to the first exacerbation after enrolment. All statistical analyses were conducted using JMP 16.1 (SAS Institute, Cary, North Carolina, USA) and R version 4.3.2 (R Foundation for Statistical Computing, Vienna, Austria). Statistical significance was set at *p* < 0.05.

## 3 Results

Figure S1 shows the flowchart of patient selection. Of 279 patients in the original cohort, 121 underwent CT using the same machine used at Hokkaido University Hospital. Of these patients, 25 were excluded for the following reasons: no CT data either at baseline or year 1 (*N* = 4), different reconstruction kernels (*N* = 19), and abnormal shadows (*N* = 2). Finally, 96 patients (male/female, 89/7) were eligible for this study.

Of 154 patients in the validation cohort, 24 were excluded due to interstitial pneumonia (*N* = 4), asthma (*N* = 2), bronchiectasis (*N* = 1), abnormal shadows (*N* = 11), history of malignancy (*N* = 5), and lobectomy due to lung cancer (*N* = 1). Finally, 130 patients (male/female: 130/0) were eligible for this study (Figure S1).

Table S2 presents the characteristics of the two cohorts. Body mass index (BMI) and several functional indices, such as percent predicted forced expiratory volume in 1 s (%FEV_1_), FEV_1_/forced vital capacity (FVC), percent predicted carbon monoxide diffusing capacity (%DLco), percent predicted transfer coefficient (%Kco), percent predicted total lung capacity (%TLC), and residual volume (RV)/TLC, were significantly higher in the original cohort than in the validation cohort.

### 3.1 Original Cohort

#### 3.1.1 Comparisons Between the High and Low AVR Groups

**FIGURE 1:**
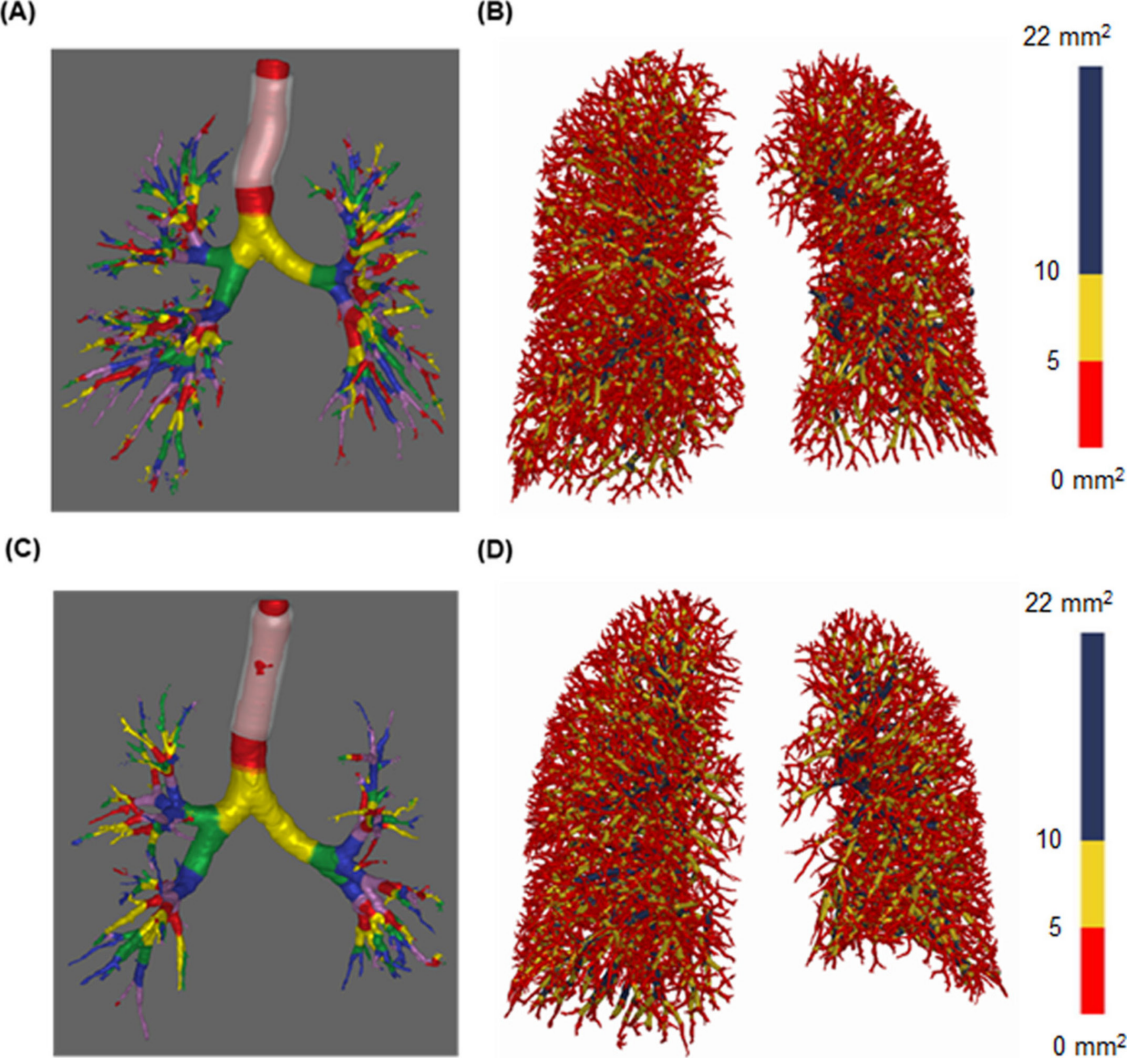
CT images of a patient with high AVR and of one with low AVR. Three dimensional images of the airways and lung vessels in patients with high AVR (A, B) and low AVR (C, D). The prolongation of the airway tree is observed in the patient with high AVR, while a less complex airway tree is observed in the patient with low AVR. (A, B) 75 years old, Male. AVR: 0.411, TAV/LV: 0.024, TBV/LV: 0.059, TAC: 332. (C, D) 63 years old, Male. AVR: 0.160, TAV/LV: 0.009, TBV/LV: 0.058, TAC: 238. AVR, volume ratio of the airway to lung blood vessels; CT, computed tomography; LV, lung volume; TAV, total airway luminal volume; TBV, total blood vessel volume; TAC, total airway count.

AVR ranged from 0.024 to 0.605 (median, 0.199), as assessed from the CT data (Figure S2A). The threshold for the highest quartile was 0.296. Representative cases are shown in Figure 1: the upper panel with high AVR (0.411) and lower panel with low AVR (0.160). The high AVR group (*N* = 24) included more patients with mild to moderate COPD than the low AVR group (*N* = 72). The high AVR group exhibited lower %RV and RV/TLC, with no differences in eosinophil levels or LV (Tables 1 and S3). TAV/LV, LA/body surface area, mReiff score, and total airway count were higher, and %WA and TBV/LV were lower in this group. PA/Ao, BV5/TBV, and %LAV did not differ significantly between the groups. AVR was lower in the group with advanced GOLD classification than in that with mild disease (Figure S3A).

**TABLE 1:** Comparisons of high and low AVR groups in the original cohort.

Original cohort	High AVR	Low AVR	*p*
*N*	24	72	
Female, *N* (%)	1 (4.17)	6 (8.33)	0.676
Age, years	70.46 (7.44)	69.49 (8.16)	0.607
Height, cm	165.28 (4.78)	161.83 (7.70)	0.043
BMI, kg/m^2^	22.37 (3.02)	22.75 (3.16)	0.604
GOLD stage, *N* (%)			0.005
1	14 (58.3)	12 (16.7)	
2	7 (29.2)	42 (58.3)	
3	2 (8.3)	17 (23.6)	
4	1 (4.2)	1 (1.4)	
Pack‐years	62.90 (33.49)	60.77 (25.82)	0.747
Ex‐smoker, *N* (%)	17 (70.8)	55 (76.4)	0.586
Chronic Bronchitis, *N* (%)	3 (12.5)	7 (9.7)	0.707
SGRQ	25.58 (17.16)	31.00 (17.02)	0.181
Exacerbation, /year	0.22 (0.35)	0.09 (0.27)	0.065
FEV_1_ Decline, mL/year	−26.55 (23.95)	−28.75 (22.20)	0.681
White blood cells, 10^3^/μL	7.0 (1.8)	6.4 (1.7)	0.145
Neutrophils, 10^3^/μL	4.3 (1.5)	3.7 (1.3)	0.057
Lymphocytes, 10^3^/μL	1.9 (0.7)	2.0 (0.7)	0.562
Monocytes, 10^3^/μL	0.56 (0.20)	0.45 (0.14)	0.005
Eosinophils, 10^3^/μL	0.19 (0.14)	0.20 (0.15)	0.802
Neutrophil‐to‐lymphocyte ratio	2.8 (2.7)	2.1 (1.1)	0.091
%FVC, %	101.25 (14.94)	101.75 (14.65)	0.885
%FEV_1_, %	76.24 (20.72)	62.86 (17.92)	0.003
FEV_1_/FVC, %	60.04 (12.72)	49.50 (11.45)	< 0.001
%DLco, %	84.53 (23.26)	86.58 (20.46)	0.682
%Kco, %	75.77 (19.82)	76.55 (22.53)	0.881
LV, L	5.33 (0.93)	5.27 (1.14)	0.828
3rd LA/BSA	35.66 (11.81)	22.72 (10.67)	< 0.001
3rd %WT, %	35.30 (2.69)	38.99 (3.87)	< 0.001
3rd %WA, %	58.53 (3.59)	62.89 (5.17)	< 0.001
%LAV, %	19.70 (13.19)	22.14 (12.30)	0.410
PA/Ao	0.80 (0.12)	0.77 (0.12)	0.175
BV5/TBV	0.52 (0.07)	0.52 (0.06)	0.983
mReiff score	2.04 (1.81)	0.46 (0.77)	< 0.001
Mucus score	1.5 (2.8)	2.3 (3.5)	0.277
TAC	353.2 (51.8)	241.9 (63.7)	< 0.001
TAV/LV	0.02 (0.01)	0.01 (0.00)	< 0.001
TBV/LV	0.05 (0.01)	0.06 (0.01)	0.013

*Note*: Data are shown as the mean (SD) and number (%). Comparisons between the groups were conducted using Student's *t*‐test, *χ*2 test, or Fisher's exact test.

Abbreviations: AVR, volume ratio of the airway to lung blood vessels; BMI, body mass index; BV5, aggregate blood vessel volume of < 5 mm^2^ in the lungs; %DLco, percent predicted carbon monoxide diffusing capacity; FEV_1_, forced expiratory volume in 1 s; %FEV_1_, percent predicted FEV_1_; FVC, forced vital capacity; %FVC, percent predicted FVC; GOLD, Global Initiative for Chronic Obstructive Lung Disease; %Kco, percent predicted transfer coefficient; LA, airway luminal area; %LAV, percentage of low attenuation volume; LV, lung volume; mReiff score, modified Reiff score; PA/Ao, ratio of the pulmonary artery diameter to aorta diameter; SD, standard deviation; SGRQ, St. George's Respiratory Questionnaire; TAC, total airway count; TAV, total airway luminal volume; TBV, total blood vessel volume; %WA, percentage of wall area; %WT, percentage of wall thickness.

#### 3.1.2 Relationships Between AVR and CT Indices, mReiff Score, and Airway Indices

**FIGURE 2:**
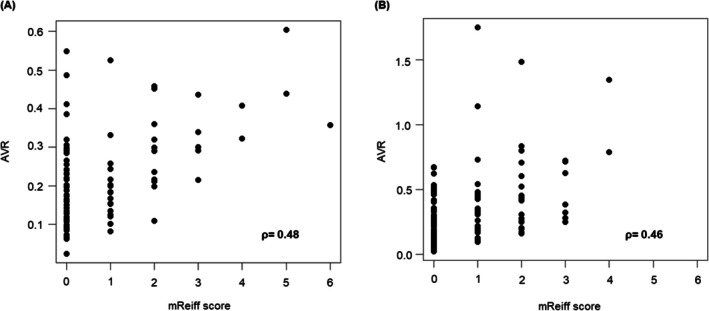
Correlations between the mReiff score and AVR in the two cohorts. A weak positive correlation was found between the mReiff score and AVR in both the original (A) and validation (B) cohorts. mReiff score, modified Reiff score; AVR, volume ratio of the airway to lung blood vessels.

Figure 2A shows the positive correlation between AVR and mReiff scores. Rho correlation coefficients were 0.48 (*p* < 0.001). Notably, AVR varied among patients in a group with an mReiff score of 0, indicating no visual bronchiectasis. A very weak correlation was observed between the mReiff score and LA or %WA (Figure S4).

#### 3.1.3 Relationships Between AVR and Exacerbation (Kaplan–Meier Curves and Multivariate Analysis)

**FIGURE 3:**
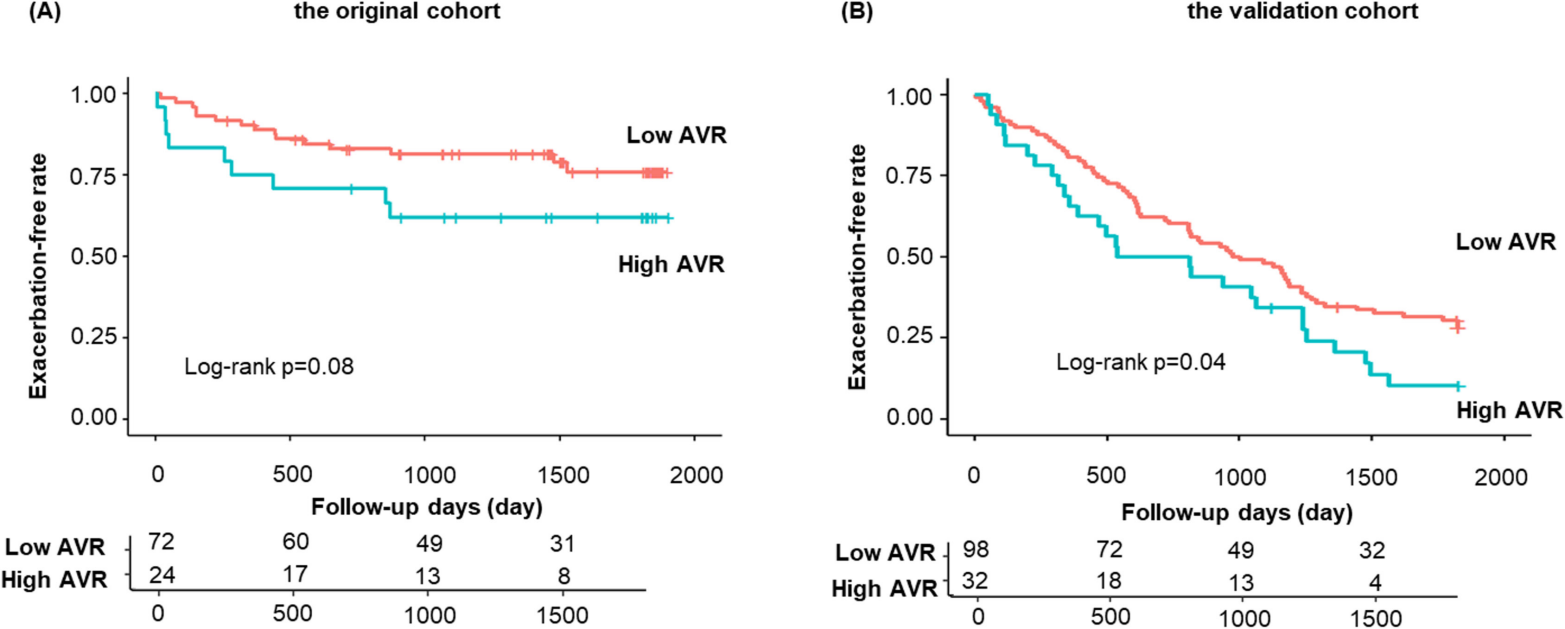
Time to the first exacerbation in the high and low AVR groups during the 5‐year follow‐up period in the two cohorts. The high AVR group showed a shorter time to first exacerbation than the low AVR group during the 5‐year follow‐up period in the original (A) and validation (B) cohorts. AVR, volume ratio of the airway to lung blood vessels.

During the 5‐year observational period, nine first events were observed in 24 patients in the high AVR group, and 15 first events in 72 patients in the low AVR group. The high AVR group showed a shorter time to first exacerbation during the 5‐year observational period than the low AVR group (Figure 3A). In the multivariate analysis, high AVR was associated with the incidence of first exacerbation, even after adjusting for smoking status, pack‐years, %FEV_1_, the mucus score, and the mReiff score (Model 1), accompanied by PA/Ao, %WA, and %LAV (Model 2) or PA/Ao, %WA, and %Kco (Model 3) (Table 2A). During the 1‐year observational period, six first events were observed in 24 patients in the high AVR group, and seven first events in 72 patients in the low AVR group. The high AVR group showed a shorter time to first exacerbation during the 1‐year observational period (Figure S5). Table S4 shows the comparisons between the patients with mReiff scores of 0 (*N* = 56) and > 0 (*N* = 40). There were no significant differences in the anthropological data or pulmonary function test values, except for pack‐years of tobacco smoking. TAV and TAV/LV were lower in patients with an mReiff score of 0 than in those with an mReiff score > 0. Among patients with an mReiff score of 0, four first events were observed in seven patients in the high AVR group, and eight first events in 49 patients in the low AVR group during the 5‐year observational period. Notably, the high AVR group showed a significantly shorter time to first exacerbation during the 5‐year follow‐up than the low AVR group in the subgroup with no visual bronchiectasis (mReiff score = 0) (Figure S6).

**TABLE 2:** Multivariable Cox proportional hazards models for the associations between AVR and the time to first exacerbation during the 5‐year follow‐up period in the two cohorts.

(A)	Model 1		Model 2		Model 3	
Original	HR [95% CI]	*p*	HR [95% CI]	*p*	HR [95% CI]	*p*
High AVR	4.20 [1.43–12.9]	0.01	4.01 [1.25–12.9]	0.02	3.85 [1.17–12.6]	0.03
%FEV_1_ per10%	0.67 [0.50–0.89]	< 0.01	0.68 [0.49–0.91]	0.01	0.68 [0.50–0.90]	< 0.01
mReiff score per1	1.09 [0.75–1.53]	0.64	1.09 [0.75–1.54]	0.63	1.10 [0.76–1.55]	0.6
Mucus score per1	1.06 [0.94–1.17]	0.32	1.05 [0.93–1.17]	0.36	1.05 [0.93–1.17]	0.35
PA/Ao per0.1	—		1.15 [0.78–1.66]	0.46	1.15 [0.78–1.67]	0.46
%WA per1%	—		1.00 [0.92–1.10]	0.93	0.99 [0.91–1.09]	0.90
%LAV per10%	—		1.01 [0.69–1.47]	0.96	—	
%Kco per10%	—		—		0.97 [0.79–1.19]	0.77

(B)	Model 1		Model 2		Model 3	
Validation	HR [95% CI]	*p*	HR [95% CI]	*p*	HR [95% CI]	*p*
High AVR	2.05 [1.24–3.39]	0.01	1.81 [1.00–3.26]	0.05	2.01 [1.15–3.52]	0.01
%FEV_1_ per10%	0.78 [0.70–0.88]	< 0.01	0.81 [0.71–0.94]	< 0.01	0.79 [0.69–0.89]	< 0.01
mReiff score per1	1.02 [0.82–1.26]	0.86	0.99 [0.79–1.22]	0.93	1.01 [0.80–1.24]	0.96
Mucus score per1	1.05 [0.96–1.13]	0.24	1.05 [0.96–1.14]	0.23	1.05 [0.96–1.13]	0.27
PA/Ao per0.1	—		0.94 [0.79–1.12]	0.48	0.93 [0.78–1.11]	0.43
%WA per1%	—		1.01 [0.97–1.04]	0.78	1.01 [0.97–1.04]	0.77
%LAV per10%	—		1.12 [0.91–1.39]	0.28	—	
%Kco per10%	—		—		0.98 [0.89–1.08]	0.72

*Note*: Multivariate Cox proportional hazards models were employed to assess the impact of covariates, including AVR, smoking status, pack‐years, %FEV_1_, the mucus score, and the mReiff score (Model 1), accompanied by PA/Ao, %WA, and %LAV (Model 2) or PA/Ao, %WA, and %Kco (Model 3), on the time to the first exacerbation after enrolment in the original cohort (A) and validation cohort (B).

Abbreviations: AVR, volume ratio of the airway to lung blood vessels; CI, confidence interval; %FEV_1_, percent predicted forced expiratory volume in 1 s; FEV_1_, forced expiratory volume in 1 s; HR, hazard ratio; %Kco, percent predicted transfer coefficient; %LAV, percentage of low attenuation volume; mReiff score, modified Reiff score; PA/Ao, ratio of the pulmonary artery diameter to aorta diameter; TAV, total airway luminal volume; TBV, total blood vessel volume; %WA, percentage of wall area.

#### 3.1.4 Relationships of TAV/LV and TBV/LV With Exacerbation

**FIGURE 4:**
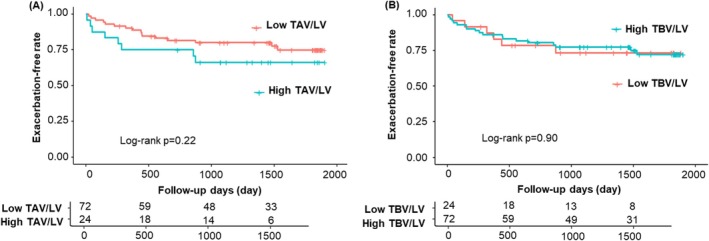
Time to first exacerbation in the high and low TAV/LV and TBV/LV groups during the 5‐year follow‐up period in the original cohort. There was no significant difference between the high and low TAV/LV (A) or TBV/LV (B) groups in the time to first exacerbation during the 5‐year follow‐up period in the original cohort. LV, lung volume; TAV, total airway luminal volume; TBV, total blood vessel volume.

There was no difference in the time to first exacerbation between the high and low TAV/LV and TBV/LV groups (Figure 4).

### 3.2 Validation Cohort

Overall, the analysis was replicated successfully. The AVR ranged from 0.025 to 1.753 (median, 0.228), as assessed based on CT data (Figure S2B); the highest quartile threshold was 0.423. BMI was lower in the high AVR group, while the spirometry values were not significantly different between the high and low AVR groups. Compared with the low AVR group (*N* = 98), the high AVR group (*N* = 32) had significantly higher TAV/LV, LA/body surface area, mReiff score, and %LAV and lower %WA, BV5/TBV, and TBV/LV (Table S5). No significant differences were seen in eosinophil levels, LV, or PA/Ao.

Figure 2B shows the positive correlation between AVR and the mReiff score. Rho correlation coefficients were 0.46 (*p* < 0.001). No statistically significant difference in AVR was observed across the GOLD classification (Figure S3B). Notably, AVR varied among patients in a group with an mReiff score of 0, which was concordant with the findings in the original cohort. Patients with an mReiff score > 0 (*N* = 60) were older and had a lower BMI, preserved St. George's Respiratory Questionnaire (SGRQ) score, and lower %Kco than those with an mReiff score of 0 (*N* = 70) (Table S6).

During the 5‐year observational period, 28 first events were observed in 32 patients in the high AVR group, while 70 first events were observed in 98 patients in the low AVR group. The high AVR group showed a shorter time to first exacerbation during the 5‐year observational period than the low AVR group (Figure 3B). On multivariate Cox proportional hazards analysis, high AVR was significantly associated with shorter time to first exacerbation across all models (Table 2B).

## 4 Discussion

This study showed that the volume mismatch between airway and lung vessel volume weakly correlated with visually assessed bronchiectasis and varied even among patients in the subgroup without visual bronchiectasis. High AVR was associated with a shorter time to first exacerbation, regardless of the mucus score, the mReiff score, PA/Ao, and %FEV_1_. Notably, a single pulmonary morphological finding, such as TAV/LV or TBV/LV, was not associated with exacerbations. To our knowledge, this study is the first to show correlations between relatively high volume ratios of the airway to lung blood vessels and exacerbation using a replication cohort, regardless of visual bronchiectasis and PA/Ao.

The disproportion between the airway volume and lung vessel volume was significantly related to time to the first exacerbation during the 5‐year follow‐up period in patients with COPD. A high AVR involves two pathologies: relatively high TAV and relatively low TBV. Despite preserved airflow and similar emphysema levels, patients with high AVR had lower %DLco than those with low AVR, suggesting impaired alveolar‐capillary diffusion and/or ventilation‐perfusion mismatch [31, 32]. However, a decrease in blood vessel volume relative to airway volume may play a role in the pathophysiology of exacerbations in a subtype of patients with COPD. Vessel morphology may be associated with poor presentation or prognosis in patients with COPD. BV5/TBV, which may reflect the pruning of the small vessels, is associated with impaired SGRQ scores [28] and spirometric severity of COPD [33].

AVR may reflect a pathophysiology different from that of visual bronchiectasis. Diaz et al. demonstrated that a deep learning‐based high volume ratio of the airway relative to the accompanying arteries correlated with exacerbations in patients with COPD [34]. In this study, a high airway‐to‐vessel volume ratio—including both arteries and veins—was associated with exacerbations, independent of the mReiff score. This suggests that a global mismatch between airways and pulmonary vessels contributes to physiological impairment beyond what regional disease explains. Notably, even in the subgroup analysis of participants with no visual bronchiectasis, AVR varied and was associated with a shorter time to the first exacerbation. Infections and ciliary motility dysfunction are key contributors to bronchiectasis [10, 11, 35]. In contrast, a high AVR may reflect inherent or acquired features of COPD. The clinical significance of a high AVR should be determined in a large‐scale study of COPD. Furthermore, the aetiology of the volume mismatch between the airway and lung vessel volume should be determined using long‐term observational cohorts.

The association of AVR with exacerbation was complementary to that of PA/Ao, which is a well‐known predictor of exacerbation and mortality in patients with COPD [13, 36]. There was no correlation between PA/Ao and TBV (data not shown), and none of the participants in this study were diagnosed with pulmonary hypertension. A lower prevalence and impact of cardiovascular diseases on mortality have been reported in Japanese patients with COPD [37, 38]. Additionally, the role of PA/Ao in exacerbations has been inconsistent among Japanese patients [39, 40]. Future studies should explore the role of AVR in exacerbation across ethnicities.

A recent study showed that an increased ratio of blood vessel volume, including arteries and veins, relative to airway volume may be related to emphysema development in patients with COPD during a 1‐year observation period [41]. However, patients with a relatively low ratio of blood vessel volume to airway volume were prone to exacerbation in this study. This finding might have been affected by the duration of the observation period and different phases of COPD. Emphysema assessed on CT was more severe in this study than in the previous study and correlated with AVR in the validation cohort but not in the original cohort, resulting in the differential impact of AVR on exacerbation (Table 2). The combined assessment of morphology and haemodynamics would broaden our insight into the interaction between the airway, parenchyma, and blood vessel alterations in respiratory diseases, including COPD.

An association between AVR and exacerbations was observed, independent of FEV_1_. Indeed, patients with high AVR showed less severe airflow limitations. This emphasises the clinical relevance of identifying subgroups with high AVR in the less severe GOLD classification. In the Kaplan–Meier analysis for exacerbations, there was no significant difference between the groups defined by the values of TAV/LV or TBV/LV. Thus, a smaller airway volume does not increase the risk of exacerbation if the blood vessel volume matches the airway size in the lungs. Conversely, it should be elucidated whether the volume mismatch between the airway and lung vessel volume, rather than lower vessel volume itself, may cause or initiate exacerbations in patients with COPD.

AVR may help define a novel COPD subtype with unique predisposition or progression patterns. One proposed trait, dysanapsis—characterised by relatively small airways compared to lung volume—is a known risk factor for COPD. Although the pathogenesis of increased AVR remains unclear, further studies are needed to investigate its onset and progression across respiratory diseases. This study identified high AVR as a potential marker of poor outcomes in COPD and suggests that it operates through mechanisms distinct from those of regional bronchiectasis. While high AVR and bronchiectasis may overlap, as indicated by weak correlations in two independent cohorts, the complementary nature of AVR to visual bronchiectasis supports a distinct underlying process. As with other imaging studies, these findings are hypothesis‐generating. Longitudinal studies and mechanistic research are needed.

This study had several limitations. First, participants of this study were Japanese and predominantly male, which fairly limits the generalisability of its results, including the AVR cut‐off. Thus, a future study should identify the validity of this study's findings using a balanced sex distribution across different ethnicities. Notably, patients in the validation cohort had more severe disease than those in the original cohort, potentially influencing exacerbation rates. Replicating these results could support broader use of AVR as a CT‐derived predictor. Second, the reconstruction kernel differed between the original and validation cohorts, which possibly affected the AVR measurement and results; therefore, further investigation is required to evaluate the role of AVR. To assess the impact of this difference on quantitative CT analysis, we tested two kernels: B30f and B60f. We found that TAV tended to be larger in CT images based on the B60f kernel than in those based on B30f, while TBV showed similar values for both kernels. Consequently, we analysed the two cohorts separately. Despite variations in measurements, we demonstrated that high AVR was associated with a shorter time to first exacerbation in both cohorts, even after adjusting for established CT‐derived indices for exacerbations, such as the visual bronchiectasis score [10, 15] and PA/Ao [13]. Third, this analysis was performed in a retrospective manner using patients from prospective studies. Thus, this study was conducted without a predetermined sample size calculation, and the sample size for the analysis might have been insufficient. However, the findings were successfully replicated in two independent cohorts, demonstrating the robustness of the results. Based on these findings, further prospective studies with a feasible and predetermined sample size are warranted. Fourth, we lacked information regarding bacterial colonisation and biomarkers in the lungs; such data could enhance our understanding of the pathophysiology underlying the volume mismatch between airway and lung vessel volume, as well as exacerbation mechanisms. Fifth, medication may influence exacerbation pathology, suggesting that results should be verified in populations treated with dual or triple inhalers [42]. Finally, we lacked data on the frequency of exacerbations in the previous year within our cohorts. History of exacerbations is recognised as a predictor of subsequent exacerbations. Therefore, the effect size of AVR on exacerbation should be assessed in conjunction with the history of exacerbations.

In conclusion, this morphological study suggests that a relative increase in airway volume to blood vessel volume in the lungs might be involved in the associated pathology of exacerbation in patients with COPD. Notably, exacerbation may develop via a differential pathway linked to the volume mismatch between the airway and lung vessel volume from that of regional bronchiectasis or extrapulmonary vessel alterations. The impact of the volume mismatch between the airway and lung vessel volume on the initiation or progression of COPD should be investigated in future studies.

## Author Contributions

**Nobuyasu Wakazono:** conceptualization (equal), conceptualization (equal), data curation (equal), data curation (equal), formal analysis (lead), investigation (lead), investigation (lead), methodology (equal), software (lead), visualization (lead), visualization (lead), writing – original draft (equal). **Kaoruko Shimizu:** conceptualization (equal), investigation (supporting), methodology (equal), writing – original draft (equal). **Naoya Tanabe:** investigation (supporting), validation (equal), writing – original draft (supporting). **Akira Oguma:** data curation (equal), investigation (supporting), software (supporting). **Hironi Makita:** investigation (supporting), supervision (equal), writing – review and editing (equal). **Kazufumi Okada:** methodology (supporting). **Miho Wakazono:** investigation (supporting). **Hiroki Nishimura:** investigation (supporting). **Yuichi Kojima:** investigation (supporting). **Michiko Takimoto‐Sato:** investigation (supporting). **Munehiro Matsumoto:** investigation (supporting). **Yuki Abe:** investigation (supporting). **Ayako Igarashi‐Sugimoto:** investigation (supporting). **Nozomu Takei:** investigation (supporting), software (supporting). **Hirokazu Kimura:** investigation (supporting), supervision (equal). **Houman Goudarzi:** investigation (supporting), supervision (equal). **Takeshi Hattori:** investigation (supporting), supervision (equal). **Ichizo Tsujino:** investigation (equal), supervision (equal), writing – review and editing (equal). **Susumu Sato:** investigation (supporting), supervision (equal), validation (equal), writing – review and editing (equal). **Shigeo Muro:** funding acquisition (equal), investigation (supporting), project administration (equal), supervision (equal), validation (equal), writing – review and editing (equal). **Masaharu Nishimura:** funding acquisition (equal), investigation (supporting), project administration (equal), resources (equal), supervision (equal), writing – review and editing (equal). **Toyohiro Hirai:** investigation (supporting), project administration (equal), validation (equal), writing – review and editing (equal). **Satoshi Konno:** funding acquisition (equal), investigation (supporting), project administration (equal), resources (equal), supervision (equal), writing – review and editing (equal).

## Disclosure

This research was previously presented at the 2024 Congress of the American Thoracic Society (ATS).

## Ethics Statement

This study was conducted according to the principles of the Declaration of Helsinki and the Ethical Guidelines for Medical and Health Research Involving Human Subjects (Japanese Ministry of Health, Labor and Welfare). The Hokkaido COPD cohort study was approved by the IRB of Hokkaido University Hospital (reference number: 018‐0394). The Kyoto University cohort was approved by the Ethics Committee of Kyoto University (approval E182). Prior to the commencement of the study, all participants in the cohort provided either written or oral informed consent.

## Conflicts of Interest

The authors have reported the following to *Respirology*: K.S. was supported by grants from Daiwa Health Development Inc., outside the submitted work. Na.T. was supported by grants from FUJIFILM Co. Ltd., and Daiichi Sankyo Company Ltd., and received honoraria from AstraZeneca, outside the submitted work. A.I.‐S. was supported by grants from Bayer Yakuhin Ltd., outside the submitted work. I.T. was supported by grants from Mochida Pharmaceuticals K.K., Nippon Shinyaku Co. Ltd., Nippon Boehringer Ingelheim Co. Ltd., Medical System Network Co. Ltd., Kaneka Corp., and Takeyama Co. Ltd., and received honoraria from Nippon Shinyaku Co. Ltd., and Janssen Pharmaceutical K.K., outside the submitted work. S.M. was supported by grants from ROHTO Pharmaceutical Co. Ltd., and FUKUDA Life Tech Co. Ltd., and received honoraria from Nippon Boehringer Ingelheim Co. Ltd., AstraZeneca K.K., and GlaxoSmithKline K.K., outside the submitted work. To.H. was supported by grants from FUJIFILM Co. Ltd., and Daiichi Sankyo Co. Ltd., and received payment for lectures from Nippon Boehringer Ingelheim Co., outside the submitted work. S.S. was supported by grants from Nippon Boehringer Ingelheim Co., Philips‐Respironics, Fukuda Denshi, Fukuda Lifetec Keiji, and ResMed, outside the submitted work. S.K. was supported by grants from Mochida Pharmaceuticals K.K., Nippon Shinyaku Co. Ltd., Nippon Boehringer Ingelheim Co. Ltd., Medical System Network Co. Ltd., Kaneka Corp., Takeyama Co. Ltd., and Novartis, and received honoraria from AstraZeneca and KYORIN Pharmaceutical, outside the submitted work. None of these companies played a role in the design or analysis of the study or in the writing of the manuscript. N.W., A.O., H.M., K.O., M.W., H.N., Y.K., M.T.S., M.M., Y.A., No.T, H.K., H.G., Ta.H., and M.N. declare no conflicts of interest.

## Supporting information


**Appendix S1:** Supporting Information.

## Data Availability

All requests for raw and analysed data and materials will be reviewed by the corresponding author to verify whether the request is subject to confidentiality obligations.
