# Supplementary material for: High Airway‐To‐Vessel Volume Ratio and Visual Bronchiectasis Are Associated With Exacerbations in COPD
Source: Respirology. 2025 Aug 31;30(12):1131–40. doi: 10.1111/resp.70114 (PMC12668884; doi:10.1111/resp.70114)
Supplement: Supplementary file 1 — Appendix S1: Supporting Information. [file RESP-30-1131-s001.docx]

**SUPPORTING INFORMATION**

**High airway-to-vessel volume ratio and visual bronchiectasis are associated with exacerbations in COPD**

**Nobuyasu Wakazono, M.D.^1^, Kaoruko Shimizu, M.D., Ph.D.^1*^, Naoya Tanabe, M.D., Ph.D.^2^, Akira Oguma, M.D., Ph.D.^1^, Hironi Makita, M.D., Ph.D.^3^, Kazufumi Okada, Ph.D.^4^, Miho Wakazono, M.D.^1^, Hiroki Nishimura, M.D.^1^, Yuichi Kojima, M.D.^1^, Michiko Takimoto-Sato, M.D., Ph.D.^1 5^, Munehiro Matsumoto, M.D., Ph.D.^1^, Yuki Abe, M.D., Ph.D.^1^, Ayako Igarashi-Sugimoto, M.D., Ph.D.^1^, Nozomu Takei, M.D., Ph.D.^1^, Hirokazu Kimura, M.D., Ph.D.^1^, Houman Goudarzi, M.D., Ph.D.^1^, Takeshi Hattori, M.D., Ph.D.^6^, Ichizo Tsujino, M.D., Ph.D.^1^, Susumu Sato, M.D., Ph.D.^2^, Shigeo Muro, M.D., Ph.D.^7^, Masaharu Nishimura, M.D., Ph.D.^1 3^, Toyohiro Hirai, M.D., Ph.D.^2^, Satoshi Konno, M.D., Ph.D.^1^**

**Contents**

S1. Supplementary Methods

S2. Supplementary Tables

S3. Supplementary Figures

S4. References

**Appendix S1. Supplementary Methods**

**S1.1. Original cohort**

The Hokkaido COPD Cohort Study, a multicenter observational prospective cohort study, was initiated in 2003–2005 and was completed in 2013–2015.^E1^ ^E2^ The diagnosis of chronic obstructive pulmonary disease (COPD) was confirmed by a respirologist before enrollment in the study. Patients with COPD underwent chest computed tomography (CT) scans annually during an exacerbation-free stable period for the first 5 years. We defined patients who had analyzable CT data for airway- and blood vessel-related indices obtained using the same machine either at enrollment or 1 year later as eligible for this analysis.

**S1.1.1. Clinical indices and outcomes**

Symptoms were defined as the worsening or new onset of two of the major symptoms: increased dyspnea, change in sputum purulence, or increased sputum volume. Alternatively, the presence of any one of the major symptoms plus any of the minor symptoms as follows; fever, increased cough, or wheezing compared with baseline. The presence of chronic cough and phlegm expectoration was considered when they occurred on most days for >3 months/year for >2 consecutive years. The diagnosis of chronic bronchitis was based on a sputum volume>10 ml/day.^E3^ Health-related quality of life, assessed using the St. George's Respiratory Questionnaire (SGRQ), was examined every year.^E4^ This study utilized annual changes in post-bronchodilator forced expiratory volume in 1 s (FEV_1_) calculated using a linear mixed-effects model from our previous study.^E2^

A dedicated office was established with several full-time Clinical Research Coordinators (CRCs) to monitor the worsening of the condition in target patients. To minimize memory bias, postcards were exchanged monthly, with results aggregated every six months. Patients were instructed to report any deterioration in their condition on the postcards, including the symptoms such as cough, sputum, or shortness of breath, as well as changes in prescribed antibiotics, emergency medical care, and whether hospitalization. The communication section of the postcard was designed to capture even minor changes in daily life. In cases where changes were reported or details were unclear, direct phone interviews were conducted with patients to gather further information. We aimed to thoroughly understand the actual situation, including any emergency visits to other medical institutions, to ensure that no worsening went undetected. Additionally, CRCs visited clinical research facilities and performed chart reviews to confirm the actual state of worsening and check for any undetected cases.^E1^ ^E2^

**S1.1.2. CT imaging assessment**

**S1.1.2.1 Intrapulmonary indices**

Quantitative analysis of imaging data and communications in Medicine (DICOM) data were performed using AVIEW (Coreline soft Inc., Seoul, South Korea). Airway candidate patches were extracted from sample points located at 6 voxels around the endpoints. By classifying them as airway or non-airway, the airways were prolonged and constructed. The bronchi, a part or all of which were located in the masked lungs, were recognized as the intrapulmonary bronchi. The volume ratio of the airway to lung blood vessels (AVR) was defined on CT as the ratio of total airway luminal volume (TAV) to total blood vessel volume (TBV). We used the ratio of low attenuation volume less than −950-HU to the lung volume (LV) on CT (%LAV) as an index for emphysema. The average airway luminal area (LA), wall area, and wall thickness in the central one-third of the branches were automatically measured and averaged. LA, percentage of wall area (%WA), percentage of wall thickness (%WT), the ratio of the wall area and wall thickness to the summed area between the wall and lumen at the right apical (RB1) and lateral basal (RB8) segmental airways were averaged. Then, averaged LA and WA were normalized by the body surface area.^E5^ Total airway count was determined by summing all airway segments from the segmented airway tree. The airway tree was segmented and skeletonized, resulting in defining the centerline of the tree. Branching points were identified with the generation number of each branch based on automatic tracking from the right and left main bronchus (generation =1) down to peripheral airway branches, using Python package “Skan” and a custom script with manual modification as needed.^E6 E7^

**S1.1.2.2 Visual assessment of bronchiectasis**

The Reiff scoring system comprises the site, type, extent, bronchial wall thickening, and bronchial dilation of bronchiectasis. Due to its complexity, the Reiff score is difficult to use in clinical practice. The mReiff scoring system was simpler for ensuring that extensive but not prominent bronchial dilatation was not overlooked.^E8^ Patients with severe bronchiectasis were excluded from the original cohort. The measurements of mReiff score were performed by two pulmonologists (NW and KS), and the interclass correlation coefficient was 0.72. Patients with a pre-existing diagnosis of bronchiectasis were excluded in this study.

**S1.1.2.3 Ratio of the pulmonary artery diameter to aorta diameter (PA/Ao)**

The diameters of the main pulmonary artery at the level of its bifurcation and [ascending aorta](https://www.sciencedirect.com/topics/medicine-and-dentistry/ascending-aorta) at its maximum dimension were assessed on a single slice of anonymized mediastinal images using custom software (AZE Ltd., Japan).^E9^

**S1.1.3. Pulmonary function tests**

Pulmonary function tests and CT were performed at the same visit. Spirometry, lung volumes, and diffusion capacity were assessed as carbon monoxide diffusing capacity (DLco) and predicted transfer coefficient (Kco), and DLco/alveolar volume was measured using a rolling seal Chestac-33 spirometer (Chest MI, Tokyo, Japan) according to the Japanese Respiratory Society guidelines,^E10^ which are similar to the American Thoracic Society guidelines.^E11^ ^E12^ The Burrow prediction equation was used for DLco and Kco calculations.^E13^ Total lung capacity (TLC) and residual volume (RV) were expressed as a percentage of the predicted values according to the Nishida prediction equations.^E14^

**S1.2. Validation cohort**

The Kyoto University cohort has been described elsewhere.^E15^ In total, 130 male participants underwent chest inspiratory CT scans and pulmonary function tests and were followed up for a median of 2542 days. All CT images with a 0.5 mm slice thickness at full inspiration were obtained in the Kyoto University cohort using an Aquilion 64 scanner (Toshiba, Tokyo, Japan) with 0.5 mm collimation, scan time of 500 ms, 120 kVp, and auto-exposure control; the images were reconstructed using a sharp algorithm (FC56). Spirometry, lung volume, DLco, and Kco were measured using the Chestac-65V instrument (Chest M.I. Inc., Tokyo, Japan). Predicted pulmonary function values were calculated according to the Japanese Respiratory Society guidelines. The occurrence of moderate-to-severe exacerbations requiring antibiotics and/or systemic steroids were recorded.^E16^ The airway and parenchymal indices, TBV, and aggregate blood vessel volume of <5 mm^2^ in the lungs (BV5) were also measured using AVIEW.

**S1.3. Statistical analysis**

Data were shown as mean values with standard deviations (SD) unless otherwise specified. AVR, representing skewed data, was expressed as median values with an interquartile range (IQR). Student’s t-test, χ2 test, or Fisher’s exact test was applied for group comparisons. AVR was measured at only one timepoint, and we examined whether there was a difference in the occurrence of COPD exacerbations between the high and low AVR groups not only over the 5-year observation period but also very early in the first year. Therefore, 1-year exacerbation-free survival was compared between participants with high and low AVRs using the Kaplan-Meier method with the log-rank test in the original cohort besides a 5-year analysis in both cohorts. Multivariate Cox proportional hazards models were employed to assess the impact of covariates, including AVR, smoking status, pack-years, percent predicted FEV_1_ (%FEV_1_), the mucus score, and the mReiff score (Model 1), accompanied by PA/Ao, percentage of wall area (%WA) and percentage of low attenuation volume (%LAV) (Model 2) or PA/Ao, %WA and percent predicted Kco (%Kco) (Model 3) on the time to the first exacerbation after enrollment. Given the collinearity, separate models including either %LAV or %Kco were created as the multivariable models. The subgroup analysis of patients with a mReiff score of 0, implying the absence of visual bronchiectasis, was conducted for comparison between the high and low AVR groups using the Kaplan-Meier method with log-rank test during the 5-year follow-up period in the original cohort.

**S2. Supplementary Tables**

**Table S1. Summarizing CT parameters/reconstructions**

|  | **Original cohort** | **Validation cohort** |
| --- | --- | --- |
| Scanner | Somatom plus Volume Zoom | Aquilion 64 |
| Tube current | 150 mA | Auto-exposure control |
| Tube potential | 140 kVp | 120 kVp |
| Slice thickness | 1.25 mm | 0.5 mm |
| Reconstructed kernel and assessed CT parameter | Standard algorithm (B30f);  Airway  Emphysema  Vascular  Sharp algorithm (B60f);  mReiff score | Sharp algorithm (FC56);  Airway  Emphysema  Vascular  mReiff score |

CT, computed tomography; mReiff score, modified Reiff score

**Table S2. Characteristics of the two cohorts**

|  | **Original cohort** | **Validation cohort** |
| --- | --- | --- |
| N | 96 | 130 |
| Female, N (%) | 7 (7.3) | 0 (0) |
| Age, years | 69.74 (7.97) | 71.06 (8.38) |
| Height, cm | 162.69 (7.22) | 164.42 (6.11) |
| BMI, kg/m^2^ | 22.66 (3.11) | 21.38 (2.85) |
| GOLD stage, N (%) |  |  |
| 1 | 26 (27.8) | 23 (17.7) |
| 2 | 49 (51.0) | 61 (46.9) |
| 3 | 19 (19.8) | 36 (27.7) |
| 4 | 2 (2.1) | 10 (7.7) |
| Pack-years | 61.30 (27.76) | 70.01 (38.52) |
| Ex-smoker, N (%) | 72 (75.0) | 106 (81.5) |
| Chronic Bronchitis, N (%) | 10 (10.4) | NA |
| SGRQ | 29.64 (17.13) | 27.91 (15.51) |
| White blood cells,10^3^/μl | 6.5 (1.7) | 6.9 (1.9) |
| Neutrophils,10^3^/μl | 3.8 (1.3) | 4.2 (1.7) |
| Lymphocytes,10^3^/μl | 2.0 (0.7) | 2.0 (0.6) |
| Monocytes,10^3^/μl | 0.48 (0.17) | 0.42 (0.13) |
| Eosinophils,10^3^/μl | 0.20 (0.14) | 0.21 (0.15) |
| Neutrophil-to-lymphocyte ratio | 2.3 (1.7) | 2.3 (1.1) |
| %VC, % | 94.58 (16.32) | 93.56 (17.65) |
| %FVC, % | 101.63 (14.65) | 97.00 (19.62) |
| %FEV_1_ , % | 66.20 (19.44) | 57.61 (20.29) |
| FEV_1_/FVC, % | 52.13 (12.58) | 47.24 (12.68) |
| %DLco, % | 86.07 (21.09) | 51.84 (18.43) |
| %Kco, % | 76.35 (21.78) | 63.14 (23.37) |
| V_A_, L | 4.04 (0.75) | 4.63 (0.71) |
| IC/TLC, % | 35.71 (8.65) | 37.39 (7.37) |
| %TLC, % | 111.06 (16.16) | 103.39 (14.18) |
| %FRC, % | 121.48 (25.27) | 108.71 (19.17) |
| %RV, % | 137.30 (41.17) | 132.11 (31.24) |
| RV/TLC, % | 46.00 (9.64) | 42.41 (8.33) |
| LV, L | 5.29 (1.08) | 5.35 (0.94) |
| 3^rd^ LA/BSA | 25.95 (12.27) | 28.51 (18.40) |
| 3^rd^ %WT, % | 38.07 (3.94) | 30.68 (4.03) |
| 3^rd^ %WA, % | 61.80 (5.16) | 52.27 (5.69) |
| %LAV, % | 21.53 (12.50) | 14.63 (13.33) |
| PA/Ao | 0.78 (0.12) | 0.86 (0.11) |
| BV5/TBV | 0.52 (0.06) | 0.56 (0.06) |
| mReiff score | 0.85 (1.31) | 0.76 (0.99) |
| Mucus score | 2.1(3.3) | 1.7(2.6) |
| TAC | 268.2 (77.2) | NA |
| TAV/LV | 0.01 (0.01) | 0.01 (0.01) |
| TBV/LV | 0.05 (0.01) | 0.05 (0.01) |

BMI, body mass index; BV5, aggregate blood vessel volume of <5 mm^2^ in the lungs; %DLco, percent predicted carbon monoxide diffusing capacity; FEV_1_, forced expiratory volume in 1 s; %FEV_1_, percent predicted FEV_1_; FVC, forced vital capacity; %FRC, percent predicted functional residual capacity; %FVC, percent predicted FVC; GOLD, Global Initiative for Chronic Obstructive Lung Disease; IC, inspiratory capacity; %Kco, percent predicted transfer coefficient; LA, airway luminal area; %LAV, percentage of low attenuation volume; LV, lung volume; mReiff score, modified Reiff score; NA, not available; PA/Ao, ratio of the pulmonary artery diameter to the aorta diameter; RV, residual volume; %RV, percent predicted RV; SD, standard deviation; SGRQ, St. George's Respiratory Questionnaire; TAC, total airway count; TAV, total airway luminal volume; TBV, total blood vessel volume; TLC, total lung capacity; %TLC, percent predicted TLC; V_A_, alveolar volume; %VC, percent predicted vital capacity; %WA, percentage of wall area; %WT, percentage of wall thickness

Data are presented as the mean (standard deviation (SD)) and number (%). Comparisons between the groups were conducted using Student’s t-test, χ2 test, or Fisher’s exact test.

**Table S3. Comparisons between the high and low AVR groups in the original cohort**

| **Original cohort** | **High AVR** | **Low AVR** | **p-value** |
| --- | --- | --- | --- |
| N | 24 | 72 |  |
| %VC, % | 98.26 (14.75) | 93.32 (16.74) | 0.202 |
| V_A_, L | 4.04 (0.56) | 4.03 (0.80) | 0.958 |
| IC/TLC, % | 38.91 (8.89) | 24.61 (8.35) | 0.035 |
| %TLC, % | 107.70 (11.99) | 112.20 (17.27) | 0.240 |
| %FRC, % | 115.15 (20.23) | 123.61 (26.55) | 0.157 |
| %RV, % | 122.49 (36.46) | 142.30 (41.7) | 0.041 |
| RV/TLC, % | 41.66 (9.82) | 47.46 (9.20) | 0.010 |

%FRC, percent predicted functional residual capacity; IC, inspiratory capacity; RV, residual volume; %RV, percent predicted RV; SD, standard deviation; TLC, total lung capacity; %TLC, percent predicted TLC; V_A_, alveolar volume; %VC, percent predicted vital capacity.

Data are shown as the mean (SD). Comparisons between the groups were conducted using Student’s t-test.

**Table S4. Comparisons between groups with an mReiff score of 0 and of >0 in the original cohort**

| **Original cohort** | **mReiff score=0** | **mReiff score>0** | **p-value** |
| --- | --- | --- | --- |
| N | 56 | 40 |  |
| Female, N (%) | 4 (7.1) | 3 (7.5) | 1 |
| Age, years | 70.20 (8.35) | 69.08 (7.44) | 0.499 |
| Height, cm | 162.03 (7.31) | 163.63 (7.06) | 0.288 |
| BMI, kg/m^2^ | 22.97 (3.24) | 22.21 (2.90) | 0.237 |
| GOLD stage, N (%) |  |  | 0.011 |
| 1 | 10 (17.9) | 16 (40.0) |  |
| 2 | 35 (62.5) | 14 (35.0) |  |
| 3 | 11 (19.6) | 8 (20.0) |  |
| 4 | 0 (0.0) | 2 (5.0) |  |
| Pack-years | 67.08 (29.59) | 53.21 (22.97) | 0.015 |
| Ex-smoker, N (%) | 45 (80.4) | 27 (67.5) | 0.152 |
| Chronic Bronchitis, N (%) | 6 (10.7) | 4 (10.0) | 1 |
| SGRQ | 31.22 (17.81) | 27.44 (16.09) | 0.288 |
| Exacerbation, /year | 0.08 (0.18) | 0.18 (0.40) | 0.072 |
| FEV_1_ Decline, mL/year | -28.89 (22.68) | -27.24 (22.60) | 0.726 |
| White blood cells,10^3^/μl | 6.4(1.5) | 6.7(1.9) | 0.336 |
| Neutrophils,10^3^/μl | 3.7(1.2) | 4.0(1.6) | 0.448 |
| Lymphocytes,10^3^/μl | 1.9(0.7) | 2.0(0.7) | 0.411 |
| Monocytes,10^3^/μl | 0.46(0.139 | 0.51(0.20) | 0.143 |
| Eosinophils,10^3^/μl | 0.21(0.12) | 0.18(0.17) | 0.305 |
| Neutrophil-to-lymphocyte ratio | 2.2(0.9) | 2.4(2.3) | 0.543 |
| %VC, % | 92.75 (16.25) | 97.05 (16.29) | 0.209 |
| %FVC, % | 101.31 (13.90) | 102.06 (15.80) | 0.806 |
| %FEV_1_, % | 63.62 (17.16) | 69.82 (21.96) | 0.124 |
| FEV_1_/FVC, % | 50.34 (11.07) | 54.65 (14.20) | 0.099 |
| %DLco, % | 87.33 (19.27) | 84.30 (23.54) | 0.49 |
| %Kco, % | 78.59 (22.28) | 73.22 (20.94) | 0.236 |
| V_A_, L | 3.99 (0.80) | 4.10 (0.68) | 0.447 |
| IC/TLC, % | 35.37 (8.39) | 36.17 (9.07) | 0.662 |
| %TLC, % | 110.91 (17.53) | 111.26 (14.27) | 0.916 |
| %FRC, % | 122.26 (26.56) | 120.40 (23.68) | 0.726 |
| %RV, % | 138.04 (41.99) | 136.27 (40.53) | 0.837 |
| RV/TLC, % | 46.62 (9.17) | 45.15 (10.32) | 0.466 |
| LV, L | 5.22 (1.17) | 5.37 (0.96) | 0.513 |
| 3^rd^ LA/BSA | 25.06 (13.84) | 27.21 (9.69) | 0.400 |
| 3^rd^ %WT, % | 38.57 (4.21) | 37.37 (3.46) | 0.142 |
| 3^rd^ %WA, % | 62.40 (5.72) | 60.97 (4.19) | 0.184 |
| %LAV, % | 21.65 (12.21) | 21.37 (13.05) | 0.916 |
| PA/Ao | 0.77 (0.12) | 0.78 (0.11) | 0.756 |
| BV5/TBV | 0.52 (0.05) | 0.52 (0.07) | 0.682 |
| mReiff score | 0.00 (0.00) | 2.05 (1.28) | <0.001 |
| Mucus score | 2.0 (3.4) | 2.2 (3.4) | 0.814 |
| TAC | 244.2 (65.7) | 301.5 (80.2) | <0.001 |
| TAV/LV | 0.01 (0.01) | 0.01 (0.01) | 0.005 |
| TBV/LV | 0.06 (0.01) | 0.05 (0.01) | 0.091 |

BMI, body mass index; BSA, body surface area; BV5, the aggregate blood vessel volume of <5 mm^2^ in the lungs; %DLco, percent predicted carbon monoxide diffusing capacity; FEV_1_, forced expiratory volume in 1 second; %FEV_1_, percent predicted FEV_1_; %FRC, percent predicted functional residual capacity; FVC, forced vital capacity; %FVC, percent predicted FVC; GOLD, Global initiative for Chronic Obstructive Lung Disease; IC, inspiratory capacity; %Kco, percent predicted transfer coefficient; LA, airway luminal area; %LAV, percentage of low attenuation volume; LV, lung volume; mReiff score, modified Reiff score; PA/Ao, the ratio of the pulmonary artery to aorta in diameter; RV, residual volume; %RV, percent predicted RV; SD, standard deviation; SGRQ, St. George's Respiratory Questionnaire; TAC, total airway count; TAV, total airway luminal volume; TBV, total blood vessel volume; TLC, total lung capacity; %TLC, percent predicted TLC; V_A_, alveolar volume; %VC, percent predicted vital capacity; %WA, percentage of wall area; %WT, percentage of wall thickness. Data are shown as the mean (SD), number (%). Comparisons between the groups were conducted using Student’s t-test, χ2 test, or Fisher’s exact test.

**Table S5. Comparisons between the high and low AVR groups in the validation cohort**

| **Validation cohort** | **High AVR** | **Low AVR** | **p-value** |
| --- | --- | --- | --- |
| N | 32 | 98 |  |
| Female, N (%) | 0 (0) | 0 (0) |  |
| Age, years | 72.38 (8.82) | 70.63 (8.23) | 0.309 |
| Height, cm | 163.78 (5.62) | 164.63 (6.28) | 0.496 |
| BMI, kg/m^2^ | 20.43 (2.80) | 21.69 (2.81) | 0.030 |
| GOLD stage, N (%) |  |  | 0.001 |
| 1 | 9 (28.13) | 14 (14.29) |  |
| 2 | 11 (34.38) | 50 (51.02) |  |
| 3 | 5 (15.63) | 31 (31.63) |  |
| 4 | 7 (21.88) | 3 (3.06) |  |
| Pack-years | 72.12 (37.60) | 69.32 (38.98) | 0.723 |
| Ex-smoker, N (%) | 28 (87.50) | 78 (79.59) | 0.434 |
| SGRQ | 34.58 (14.35) | 25.86 (15.35) | 0.008 |
| White blood cells,10^3^/μl | 7.0 (2.2) | 6.8 (1.8) | 0.696 |
| Neutrophils,10^3^/μl | 4.4 (2.0) | 4.2 (1.5) | 0.453 |
| Lymphocytes,10^3^/μl | 1.9 (0.6) | 2.0 (0.6) | 0.601 |
| Monocytes,10^3^/μl | 0.45 (0.15) | 0.41 (0.12) | 0.108 |
| Eosinophils,10^3^/μl | 0.20 (0.09) | 0.21 (0.16) | 0.855 |
| Neutrophil-to-lymphocyte ratio | 2.5 (1.3) | 2.3 (1.1) | 0.400 |
| %VC, % | 93.56 (22.68) | 93.56 (15.82) | 1 |
| %FVC, % | 95.06 (23.89) | 97.63 (18.11) | 0.521 |
| %FEV_1_, % | 58.16 (25.28) | 57.43 (18.53) | 0.862 |
| FEV_1_/FVC, % | 47.14 (13.38) | 47.27 (12.51) | 0.959 |
| %DLco, % | 42.19 (18.09) | 54.92 (17.53) | 0.001 |
| %Kco, % | 51.20 (22.66) | 66.96 (22.39) | 0.001 |
| V_A,_ L | 4.66 (0.55) | 4.61 (0.75) | 0.750 |
| IC/TLC, % | 36.33 (9.00) | 37.74 (6.76) | 0.347 |
| %TLC, % | 104.31 (12.20) | 103.08 (14.82) | 0.672 |
| %FRC, % | 110.04 (20.64) | 108.27 (18.75) | 0.652 |
| %RV, % | 135.60 (39.81) | 130.95 (28.00) | 0.467 |
| RV/TLC, % | 43.11 (11.02) | 42.18 (7.28) | 0.586 |
| LV, L | 5.62 (0.84) | 5.26 (0.95) | 0.063 |
| 3rd LA/BSA | 42.75 (24.80) | 23.86 (12.85) | <0.001 |
| 3rd %WT, % | 29.14 (4.00) | 31.19 (3.93) | 0.012 |
| 3rd %WA, % | 51.04 (5.68) | 54.00 (5.53) | 0.010 |
| %LAV, % | 23.08 (15.10) | 11.87 (11.50) | <0.001 |
| PA/Ao | 0.86 (0.10) | 0.86 (0.11) | 0.996 |
| BV5/TBV | 0.53 (0.06) | 0.57 (0.05) | <0.001 |
| mReiff score | 1.34 (1.18) | 0.57 (0.85) | <0.001 |
| Mucus score | 1.6 (2.6) | 1.7 (2.6) | 0.897 |
| TAV/LV | 0.03 (0.01) | 0.01 (0.01) | <0.001 |
| TBV/LV | 0.04 (0.01) | 0.06 (0.01) | <0.001 |

AVR, volume ratio of airway to lung blood vessel; BMI, body mass index; BV5, the aggregate blood vessel volume of <5 mm^2^ in the lungs; %DLco, percent predicted carbon monoxide diffusing capacity; FEV_1_, forced expiratory volume in 1 second; %FEV_1_, percent predicted FEV_1_; %FRC, percent predicted functional residual capacity; FVC, forced vital capacity; %FVC, percent predicted FVC; GOLD, Global initiative for Chronic Obstructive Lung Disease; IC, inspiratory capacity; %Kco, percent predicted transfer coefficient; LA, airway luminal area; %LAV, percentage of low attenuation volume; LV, lung volume; mReiff score, modified Reiff score; PA/Ao, the ratio of the pulmonary artery to aorta in diameter; RV, residual volume; %RV, percent predicted RV; SD, standard deviation; SGRQ, St. George's Respiratory Questionnaire; TAV, total airway luminal volume; TBV, total blood vessel volume; TLC, total lung capacity; %TLC, percent predicted TLC; V_A_, alveolar volume; %VC, percent predicted vital capacity; %WA, percentage of wall area; %WT, percentage of wall thickness

Data are shown as the mean (SD), number (%). Comparisons between the groups were conducted using Student’s t-test, χ2 test, or Fisher’s exact test.

**Table S6. Comparisons between groups with an mReiff score of 0 and of >0 in the validation cohort**

| **Validation cohort** | **mReiff score=0** | **mReiff score>0** | **p-value** |
| --- | --- | --- | --- |
| N | 70 | 60 |  |
| Female, N (%) | 0 | 0 | 1.000 |
| Age, years | 68.91 (8.21) | 73.57 (7.92) | 0.001 |
| Height, cm | 164.83 (6.09) | 163.95 (6.15) | 0.416 |
| BMI, kg/m^2^ | 22.00 (2.57) | 20.66 (3.01) | 0.007 |
| GOLD stage, N (%) |  |  | 0.193 |
| 1 | 10 (14.3) | 13 (21.7) |  |
| 2 | 34 (48.6) | 27 (45.0) |  |
| 3 | 23 (32.9) | 13 (21.7) |  |
| 4 | 3 (4.3) | 7 (11.7) |  |
| Pack-years | 67.98 (38.94) | 72.38 (38.22) | 0.519 |
| Ex-smoker, N (%) | 53 (75.7) | 53 (88.3) | 0.065 |
| SGRQ | 24.71 (14.41) | 31.50 (16.04) | 0.015 |
| White blood cells,10^3^/μl | 6.8(2.0) | 7.0(1.8) | 0.430 |
| Neutrophils,10^3^/μl | 4.2(1.7) | 4.3(1.6) | 0.668 |
| Lymphocytes,10^3^/μl | 1.9(0.6) | 2.1(0.6) | 0.097 |
| Monocytes,10^3^/μl | 0.41(0.13) | 0.42(0.13) | 0.932 |
| Eosinophils,10^3^/μl | 0.20(0.17) | 0.21(0.11) | 0.545 |
| Neutrophil-to-lymphocyte ratio | 2.4(1.2) | 2.2(1.0) | 0.499 |
| %VC, % | 92.06 (16.36) | 95.31 (19.05) | 0.299 |
| %FVC, % | 96.31 (18.40) | 97.79 (21.09) | 0.670 |
| %FEV_1_, % | 56.32 (18.31) | 59.11 (22.45) | 0.437 |
| FEV_1_/FVC, % | 47.15 (12.28) | 47.33 (13.22) | 0.936 |
| %DLco, % | 55.97 (18.14) | 47.01 (17.72) | 0.006 |
| %Kco, % | 67.81 (23.68) | 57.68 (21.96) | 0.014 |
| V_A_, L | 4.66 (0.69) | 4.59 (0.72) | 0.562 |
| IC/TLC, % | 36.72 (6.22) | 38.16 (8.49) | 0.268 |
| %TLC, % | 104.34 (13.87) | 102.30 (14.57) | 0.417 |
| %FRC, % | 110.83 (18.16) | 106.27 (20.14) | 0.179 |
| %RV, % | 134.07 (28.16) | 129.84 (34.55) | 0.446 |
| RV/TLC, % | 42.61 (7.59) | 42.19 (9.16) | 0.779 |
| Mucus score | 1.6 (2.6) | 1.7 (2.6) | 0.819 |

BMI, body mass index; %DLco, percent predicted carbon monoxide diffusing capacity; FEV_1_, forced expiratory volume in 1 second; %FEV_1_, percent predicted FEV_1_; %FRC, percent predicted functional residual capacity; FVC, forced vital capacity; %FVC, percent predicted FVC; GOLD, Global initiative for Chronic Obstructive Lung Disease; IC, inspiratory capacity; %Kco, percent predicted transfer coefficient; mReiff score, modified Reiff score; RV, residual volume; %RV, percent predicted RV; SD, standard deviation; SGRQ, St. George's Respiratory Questionnaire; TLC, total lung capacity; %TLC, percent predicted TLC; V_A_, alveolar volume; %VC, percent predicted vital capacity.

Data are shown as the mean (SD), number (%). Comparisons between the groups were conducted using Student’s t-test, χ2 test, or Fisher’s exact test.

S3. Supplementary Figures

**Figure S1.** Flow chart of participant selection in the two cohorts

Of the 279 patients in the original cohort, 121 underwent CT using the same machine at the Hokkaido University Hospital. Of these patients, 25 were excluded for the following reasons: no CT data either at baseline or year 1 (four patients), different reconstruction kernels (19 patients), and abnormal shadows (two patients). Of the 154 patients in the validation cohort, 24 were excluded due to interstitial pneumonia (four patients), asthma (two patients), bronchiectasis (one patient), abnormal shadows (11 patients), history of malignancy (five patients), and lobectomy due to lung cancer (one patient). Finally, 96 patients (male/female, 89/7) in the original cohort and 130 patients (male/female, 130/0) in the validation cohort were eligible for the analysis.

Abbreviations: CT, computed tomography


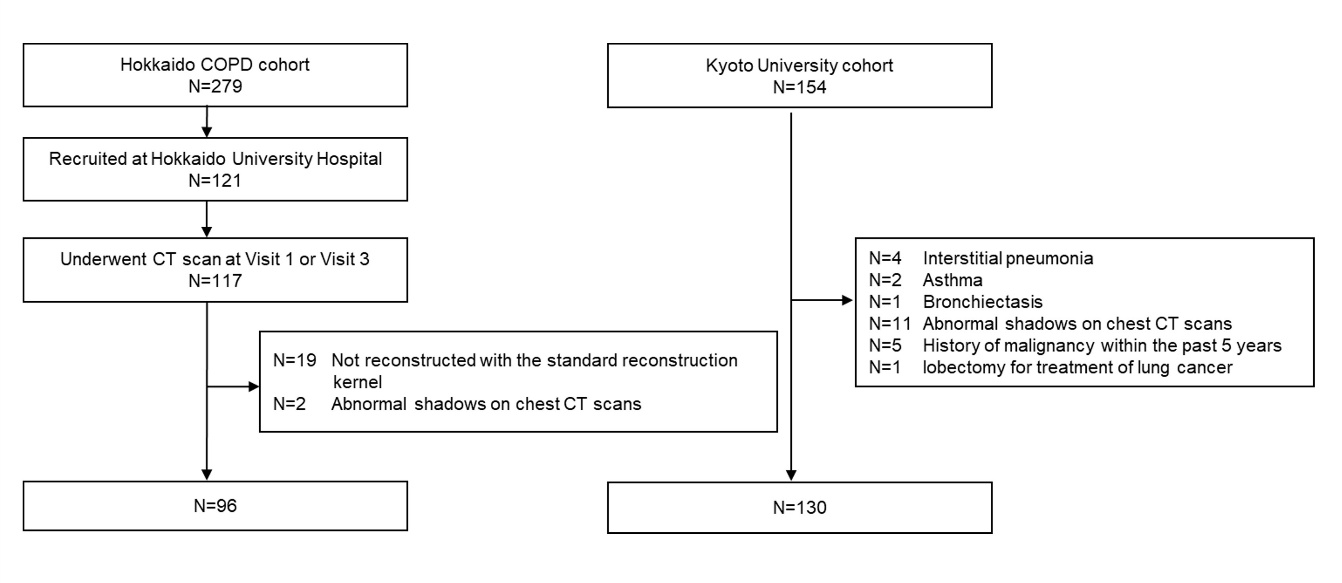


**Figure S2.** Distributions of AVR in the two cohorts

In the original cohort (A), the AVR varied from 0.024 to 0.605 (median, 0.199) as assessed based on the CT data, while the AVR ranged from 0.025 to 1.753 (median, 0.228) in the validation cohort (B). The thresholds of the highest quartiles were 0.296 and 0.423 in the original and validation cohorts, respectively.

Abbreviations: AVR, volume ratio of airway to lung blood vessel; IQR, interquartile range


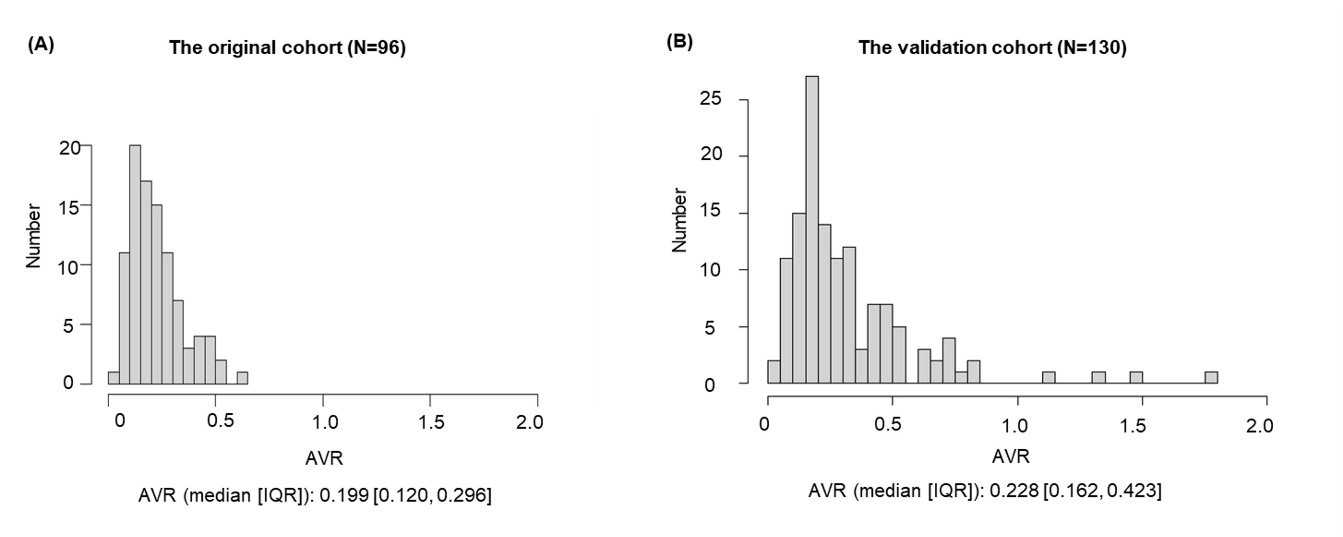


**Figure S3.** Comparison of AVR by GOLD classification

In the original cohort (A), the AVR values were indicated as follows: from 0.070 to 0.605 (median 0.298) for GOLD 1, from 0.024 to 0.487 (median 0.197) for GOLD 2, and from 0.063 to 0.408 (median 0.110) for GOLD 3 and 4. Steel test revealed that the AVR is significantly higher in GOLD 1 compared to GOLD 2 and GOLD 3 and 4. In the Validation cohort (B), the AVR values were indicated as follows: from 0.083 to 1.487 (median 0.324) for GOLD 1, from 0.059 to 0.803 (median 0.214) for GOLD 2, from 0.025 to 1.753 (median 0.220) for GOLD 3 and 4. Steel test revealed that the AVR had no statistically significant difference based on GOLD classification.

Abbreviations: AVR, volume ratio of airway to lung blood vessel; GOLD, Global Initiative for Chronic Obstructive Lung Disease


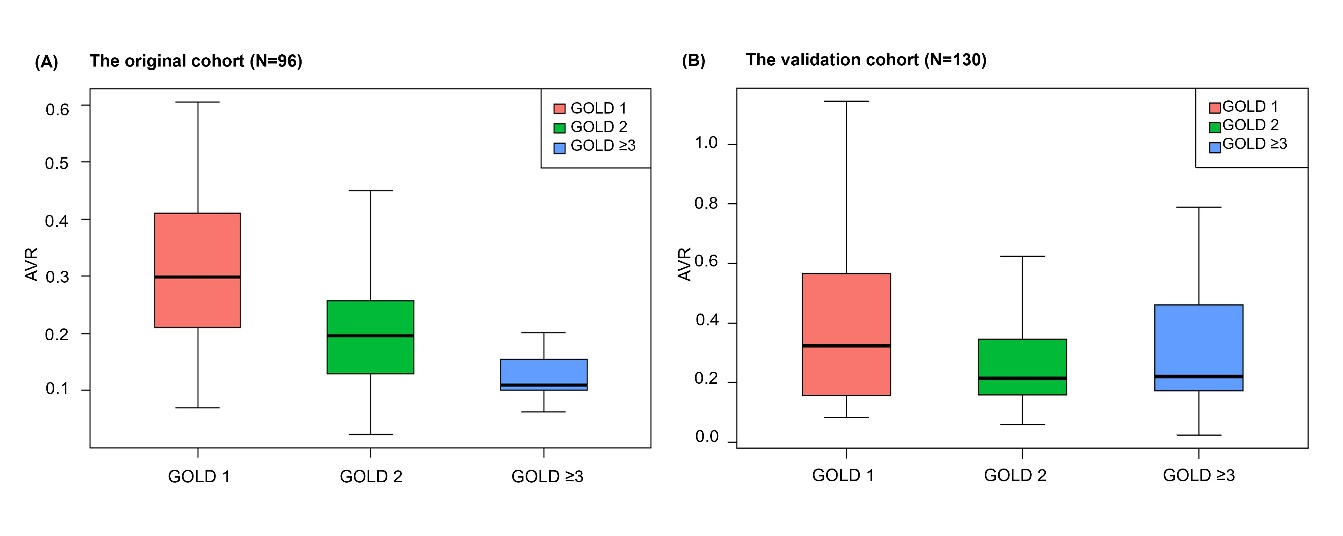


**Figure S4.** Correlations between the mReiff score and CT indexes in the original cohort

The mReiff score was very weakly correlated with LA (ρ= 0.21) and %WA (ρ= -0.22) in the original cohort. ρ-values were obtained using Spearman’s rank correlation coefficient.

Abbreviations: CT, computed tomography; LA, airway luminal area; %WA, percentage of wall area


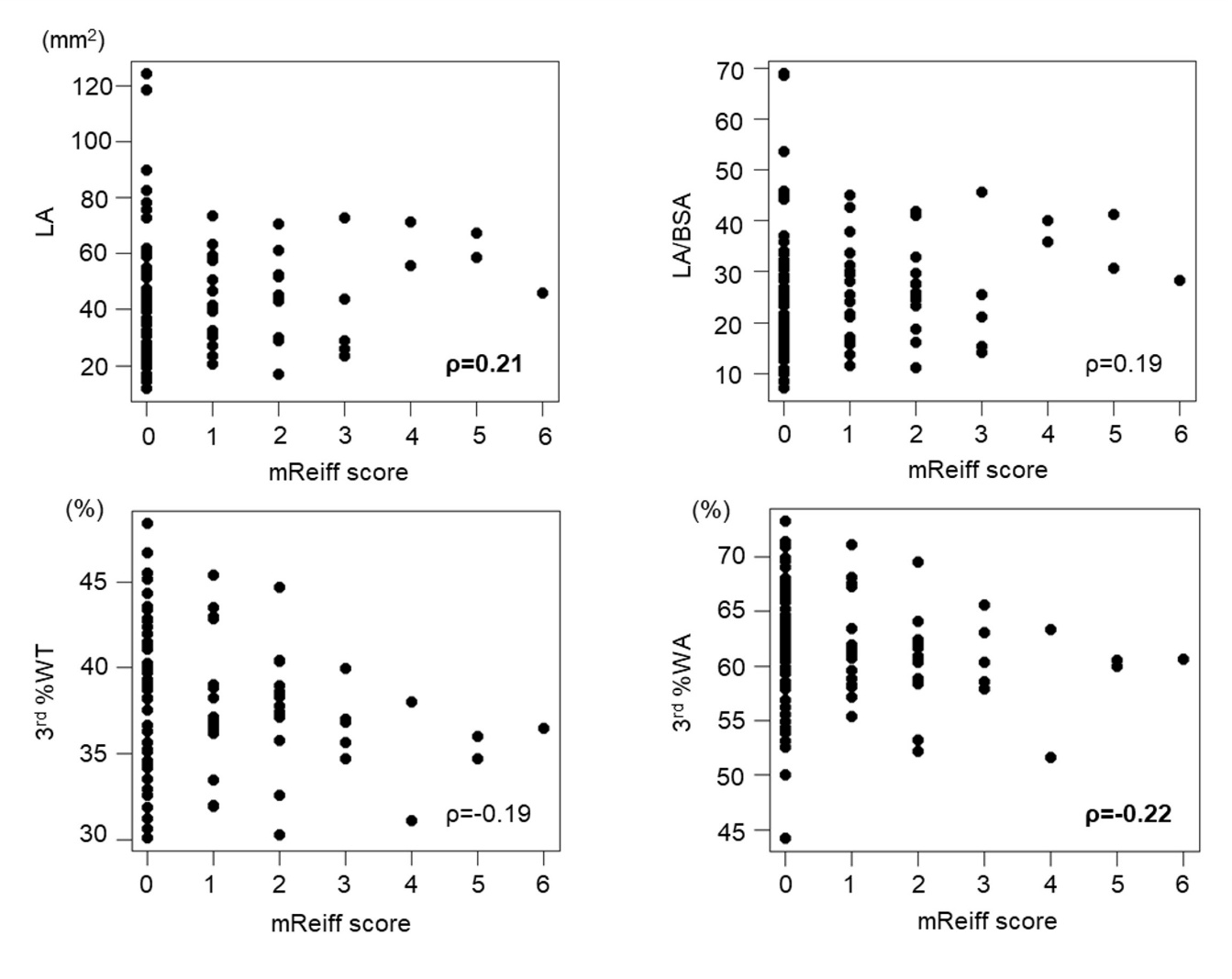


**Figure S5.** Time to first exacerbation in the high and low AVR groups during the 1-year follow-up period in the original cohort

The high AVR group experienced a shorter time to first exacerbation during the first year from baseline in the original cohort.

Abbreviations: AVR, volume ratio of airway to lung blood vessel


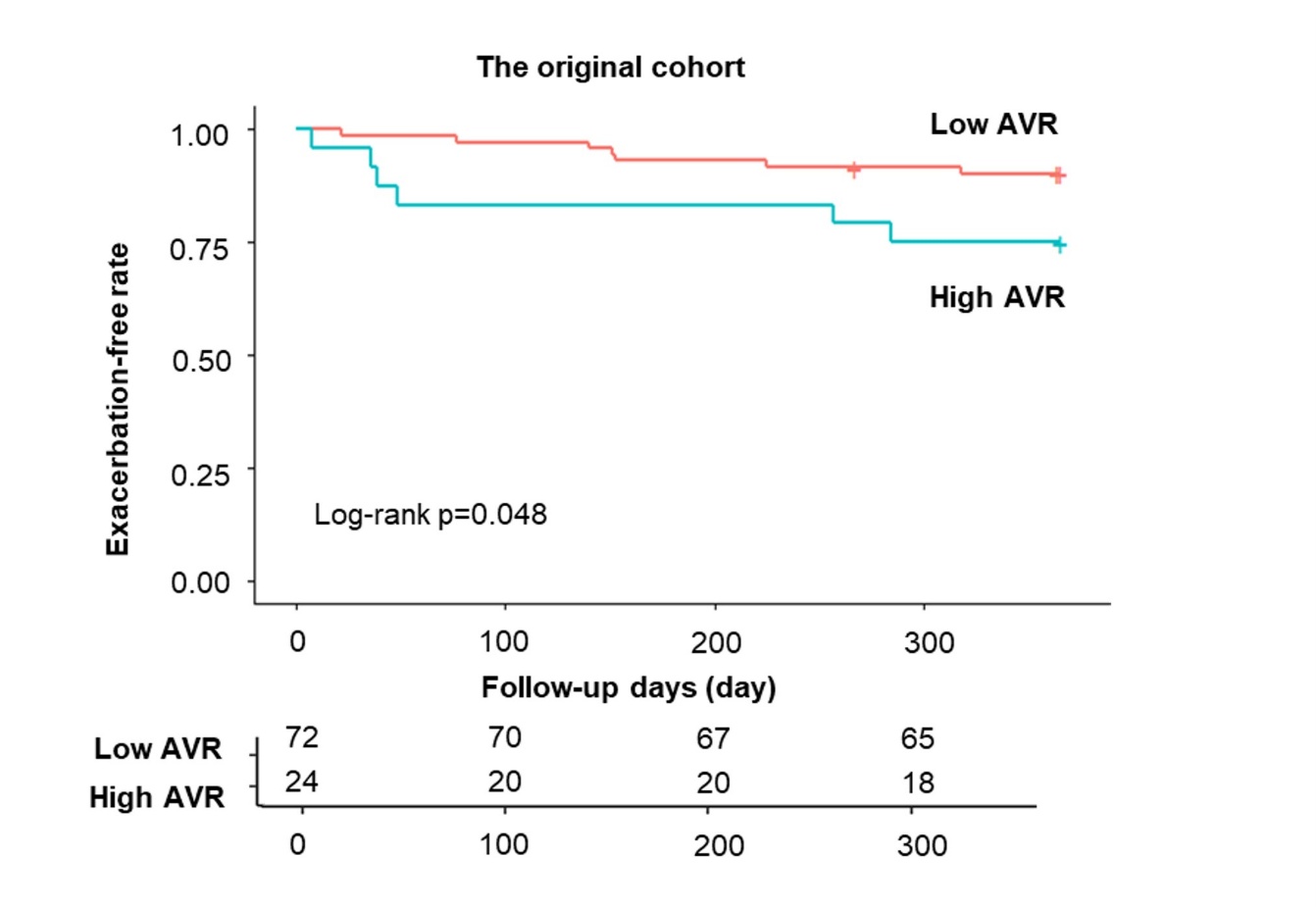


**Figure S6.** Time to first exacerbation in the high and low AVR groups with no visual bronchiectasis during the 5-year follow-up period in the original cohort

In the original cohort, the high AVR group (N=7) showed a significantly shorter time to first exacerbation during the 5-year follow-up than the low AVR group (N=49) in the subgroup with no visual bronchiectasis.

mReiff score = 0 implies the absence of visual bronchiectasis.

Abbreviations: AVR, volume ratio of airway to lung blood vessel


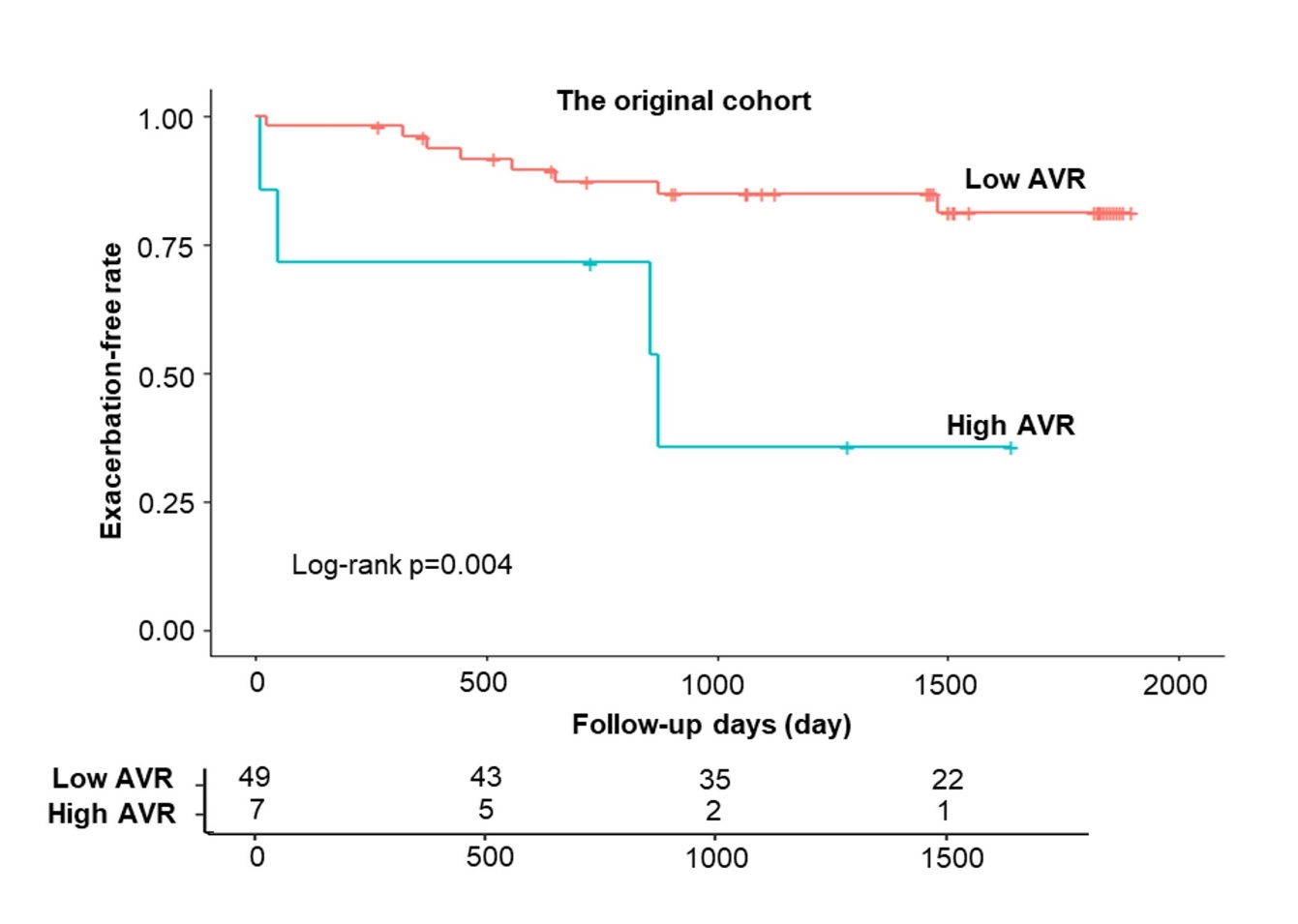


**S4. References**

E1 Makita H, Nasuhara Y, Nagai K, Ito Y, Hasegawa M, Betsuyaku T, et al. Characterisation of phenotypes based on severity of emphysema in chronic obstructive pulmonary disease. *Thorax*. 2007; **62**: 932-7.

E2 Nishimura M, Makita H, Nagai K, Konno S, Nasuhara Y, Hasegawa M, et al. Annual change in pulmonary function and clinical phenotype in chronic obstructive pulmonary disease. *Am. J. Respir. Crit. Care Med.* 2012; **185**: 44-52.

E3 Braman SS. Chronic cough due to chronic bronchitis: ACCP evidence-based clinical practice guidelines. *Chest*. 2006; **129**: 104s-15s.

E4 Jones PW, Quirk FH, Baveystock CM, Littlejohns P. A self-complete measure of health status for chronic airflow limitation. The St. George's Respiratory Questionnaire. *Am. Rev. Respir. Dis.* 1992; **145**: 1321-7.

E5 Shimizu K, Tanabe N, Oguma A, Kimura H, Suzuki M, Yokota I, et al. Parenchymal destruction in asthma: Fixed airflow obstruction and lung function trajectory. *J. Allergy Clin. Immunol.* 2022; **149**: 934-42.e8.

E6 Kirby M, Tanabe N, Tan WC, Zhou G, Obeidat M, Hague CJ, et al. Total Airway Count on Computed Tomography and the Risk of Chronic Obstructive Pulmonary Disease Progression. Findings from a Population-based Study. *Am. J. Respir. Crit. Care Med.* 2018; **197**: 56-65.

E7 Maetani T, Tanabe N, Terada S, Shiraishi Y, Shima H, Kaji S, et al. Physiological impacts of computed tomography airway dysanapsis, fractal dimension, and branch count in asymptomatic never smokers. *J Appl Physiol (1985)*. 2023; **134**: 20-7.

E8 Hatch M, Lilburn P, Scott C, Ing A, Langton D. Safety and efficacy of bronchial thermoplasty in Australia 5 years post-procedure. *Respirology*. 2023; **28**: 1053-9.

E9 Takei N, Suzuki M, Tanabe N, Oguma A, Shimizu K, Kimura H, et al. Combined assessment of pulmonary arterial enlargement and coronary calcification predicts the prognosis of patients with chronic obstructive pulmonary disease. *Respir. Med.* 2021; **185**: 106520.

E10 [Guideline of respiratory function tests--spirometry, flow-volume curve, diffusion capacity of the lung]. *Nihon Kokyuki Gakkai Zasshi*. 2004; **Suppl**: 1-56.

E11 Miller MR, Hankinson J, Brusasco V, Burgos F, Casaburi R, Coates A, et al. Standardisation of spirometry. *Eur. Respir. J.* 2005; **26**: 319-38.

E12 Graham BL, Brusasco V, Burgos F, Cooper BG, Jensen R, Kendrick A, et al. 2017 ERS/ATS standards for single-breath carbon monoxide uptake in the lung. *Eur. Respir. J.* 2017; **49**.

E13 Burrows B, Kasik JE, Niden AH, Barclay WR. Clinical usefulness of the single-breath pulmonucy diffusing capacity test. *Am. Rev. Respir. Dis.* 1961; **84**: 789-806.

E14 Nishida O, Sewake N, Kambe M, Okamoto T, Takano M. [Pulmonary function in healthy subjects and its prediction. 4. Subdivisions of lung volume in adults (author's transl)]. *Rinsho Byori*. 1976; **24**: 837-41.

E15 Terada K, Muro S, Sato S, Ohara T, Haruna A, Marumo S, et al. Impact of gastro-oesophageal reflux disease symptoms on COPD exacerbation. *Thorax*. 2008; **63**: 951-5.

E16 Tanabe N, Muro S, Hirai T, Oguma T, Terada K, Marumo S, et al. Impact of exacerbations on emphysema progression in chronic obstructive pulmonary disease. *Am. J. Respir. Crit. Care Med.* 2011; **183**: 1653-9.
